# The second “time-out”: a surgical safety checklist for lengthy robotic surgeries

**DOI:** 10.1186/1754-9493-7-19

**Published:** 2013-06-03

**Authors:** Joseph B Song, Goutham Vemana, Jonathan M Mobley, Sam B Bhayani

**Affiliations:** 1 Washington University in St. Louis School of Medicine, 4960 Children’s Place, Campus Box 8242, St. Louis, MO 63110, USA

## Abstract

Robotic surgeries of long duration are associated with both increased risks to patients as well as distinct challenges for care providers. We propose a surgical checklist, to be completed during a second “time-out”, aimed at reducing peri-operative complications and addressing obstacles presented by lengthy robotic surgeries. A review of the literature was performed to identify the most common complications of robotic surgeries with extended operative times. A surgical checklist was developed with the goal of addressing these issues and maximizing patient safety. Extended operative times during robotic surgery increase patient risk for position-related complications and other adverse events. These cases also raise concerns for surgical, anesthesia, and nursing staff which are less common in shorter, non-robotic operations. Key elements of the checklist were designed to coordinate operative staff in verifying patient safety while addressing the unique concerns within each specialty. As robotic surgery is increasingly utilized, operations with long surgical times may become more common due to increased case complexity and surgeons overcoming the learning curve. A standardized surgical checklist, conducted three to four hours after the start of surgery, may enhance perioperative patient safety and quality of care.

## Introduction

Robotic surgery has become an increasingly adopted technology. Improved dexterity in narrow spaces and faster patient recovery are just some of the advantages cited in favor of adopting this new approach. After initial applications in gynecologic and urologic surgeries, robotic operations are now becoming more common. Indeed, robotic prostatectomy is currently estimated to be the most used method for radical prostatectomy in the United States [1] and robotic surgeries are being more frequently utilized in general and thoracic surgeries [2,3].

As most practicing surgeons have not had extensive training in robotic surgery, the learning curve may be heterogeneous. Additionally, as complex operations are often embraced with robotics, longer operating room (OR) times are common in the early experience [4,5]. These prolonged operations can be a challenge on many fronts. Not only do they exacerbate surgeon fatigue, but long cases also complicate nursing and anesthetic care. Furthermore, extended cases can put the patient at increased risk for position-related patient complications such as peri-operative peripheral nerve injury and rhabdomyolysis [6,7].

Indeed, patient positioning in extended robotic operations is of particular concern in regards to patient safety. Many robotic operations involve positioning of patients in angles that may not be well studied. For example, a tendency to exaggerate the Trendelenberg position during gynecologic and urologic robotic operations has been shown to increase intraocular pressure [8]. Patient positioning is further complicated by factors which make shifts in position difficult to notice. Not only do robotic arms partially obscure the patient, but surgeons perform these operations somewhat dissociated from the patient, as they sit at a console away from the bedside. Positioning-related complications ranging from neuropathy to blindness have been reported after robotic surgery [9,10].

**Figure 1:**
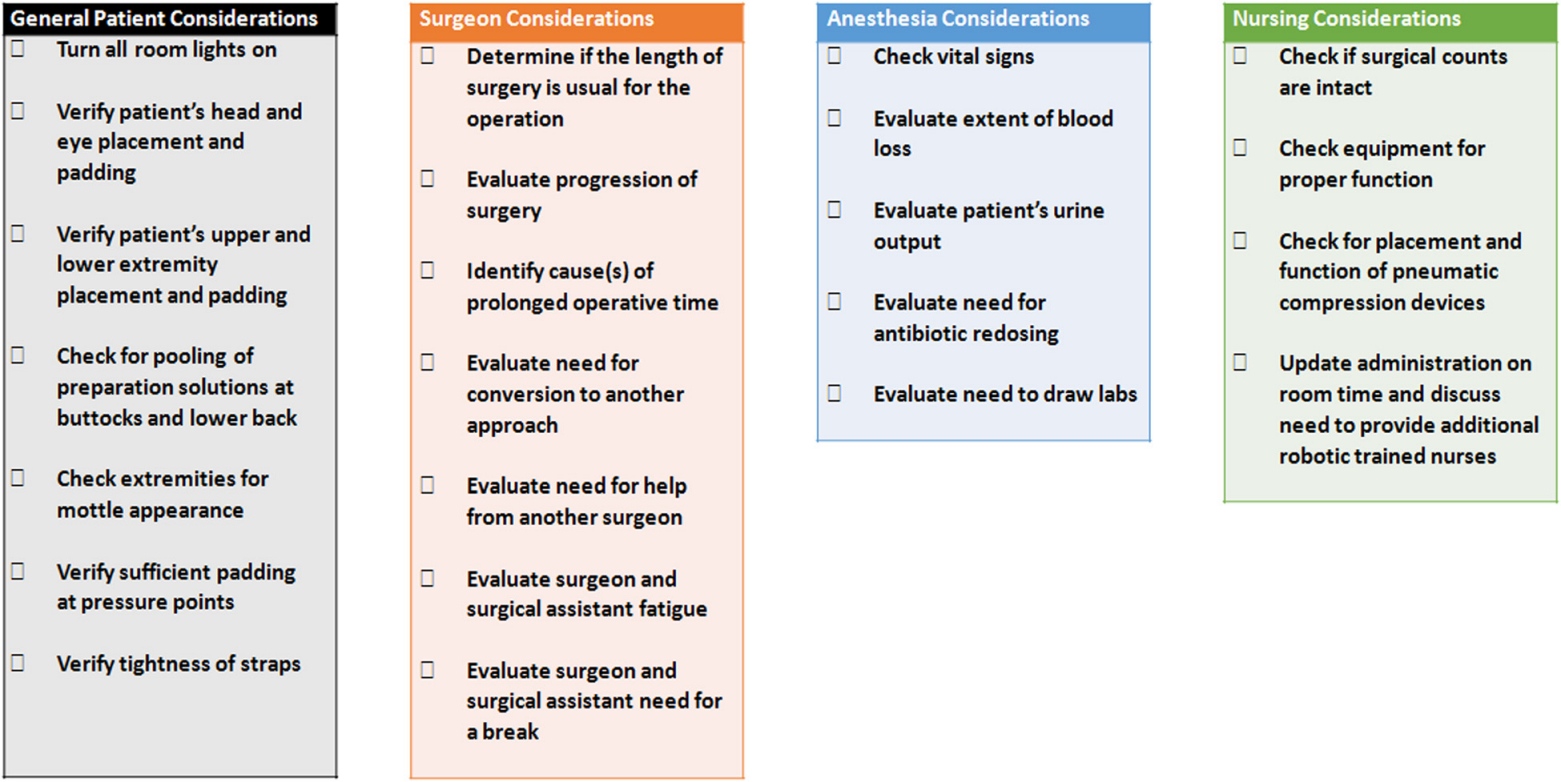
Checklist for a second time-out in extended robotic surgeries.

Complications such as those mentioned above can be challenging to address. However, since the World Health Organization published its surgical checklist in 2008, part of which includes a pre-operative “time-out”, checklists have been proven effective in decreasing perioperative morbidity and mortality [11]. To identify and possibly prevent some of the complications associated with extended robotic surgeries, we propose a “second time-out”— a checklist conducted three to four hours after the start of surgery (Figure 1). This “second time-out” is designed to assess patient safety in this unique surgical environment, particularly during the initial learning curve and with complex procedures. Furthermore, this checklist is designed to promote communication between the surgical, anesthetic and nursing staff while addressing specialty concerns which disproportionately impact patient care during prolonged robotic operations.

## Methods

Analysis of the literature was performed, particularly addressing robotic operations with prolonged OR times. Based on this review, a checklist was developed to intervene on these issues prior to them becoming adverse events. Pubmed.gov was searched for articles containing the word “prolonged”, “extended”, “time”, “duration”, “complication(s)”, “position(ing)”, “concern(s)”, “injury” or “injuries” along with the term “robotic” in the title. A total of 200 Pubmed articles fit the preliminary search criteria, of which 23 were appropriate for this study. The 23 articles reviewed were then used to further expand the search with the specific complications and concerns identified.

### Checklist components

Suggestions from the literature were scrutinized and the second time-out was developed and categorized based on general areas that should be addressed by different members of the team. Four separate areas were incorporated: general patient factors, surgeon factors, anesthesia factors, and nursing factors (Figure 1). This design allowed the checklist to be cognizant of the specialized concerns of each staff’s expertise while simultaneously engaging the entire team.

#### General patient considerations

Because robotic surgery is associated with specific positions such as steep Trendelenberg, location of the surgeon away from the operating table, and obscuration of the patient by robotic arms and extended draping, it is suggested that patient positioning be reviewed for shifts which may have gone unnoticed during long cases. This should be done with the room lights turned on, though the robot arms can remain docked and patient remain draped. All care providers should participate in reviewing patient positioning, though the need to maintain surgical sterility will limit how involved certain providers can be. Patients should be examined underneath their draping for proper extremity placement and padding. Particular attention should be paid to pressure points of the arms and legs. Any mottled appearance should be noted, which may be suggestive of rhabdomyolysis.

One of the most common results of improper patient positioning is nerve injury, which accounts for almost one third of anesthesia-related medical-legal claims in the US [12]. The effects of positioning changes are further compounded in long surgeries, as even one extra hour can significantly increase the risk of nerve damage [13]. Therefore, the second time-out serves as an invaluable opportunity to identify and prevent this potential issue.

**Figure 2:**
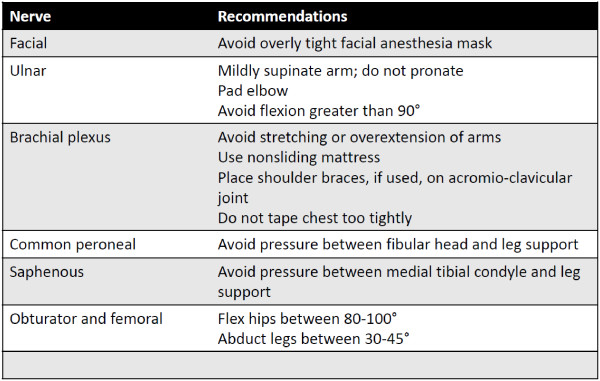
Commonly damaged nerves and recommendations on how to avoid injury.

While a full review of patient positioning is outside the scope of this paper, we have compiled a list of the most commonly injured nerves during robotic surgery (Figure 2). Firstly, commonly injured nerves in the upper extremity should be checked. This includes the ulnar nerve, which is frequently impinged when the arm is in a pronated position with the patient supine [14]. Elbow padding should be verified and, in cases where the elbow is flexed, elbow flexion should be decreased to less than 90° [15]. Furthermore, the brachial plexus should be protected by minimizing stretching and extension of arms. This is especially important in the Trendelenberg position, as cephalad shifts in position can increase pressure on the brachial plexus, especially the upper trunk [16]. In this position, the use of shoulder braces has been associated with brachial plexus injury [9]. If shoulder braces are used, it should be verified that they are placed over the acromio-clavicular joint [17,18]. Lower extremity positioning should also be re-checked, especially if the patient is in a lithotomy position. In these cases, injuries to the common peroneal and saphenous nerves are known to occur due to contact between the fibular head and medial tibial condyle, respectively, with the stirrups or leg brace [19]. Furthermore, the degree of hip flexion and angulation should be checked as improper positioning can cause injury to the obturator and femoral nerves [20]. If any signs of patient displacement are found, a break in the steep Trendelenburg positioning can allow for proper assessment and repositioning.

Non-neurologic complications of improper patient positioning can also be addressed during the second time-out. Corneal abrasions have been reported as the most frequent ocular complication in the peri-operative period [21], so proper taping of the eyes and placement and padding of the head should be verified. The tightness of straps should be re-checked for increased pressures caused by shifts in patient positioning. This is especially important in the upper and lower extremities, where over-tucking can lead to compartment syndrome [15]. The buttocks and lower back should also be check for pooling of preparation solutions, which can cause chemical burns over long periods of time [22].

#### Surgical considerations

In extended cases, surgeons may become out of sync with operative time. This is especially true in robotic surgeries, where the physician is seated comfortably, and without the bedside presence. Indeed, laparoscopic cases have been shown to result in less muscle fatigue than open surgeries [23] and robotic cases are less likely than other procedures to result in pain, numbness, or fatigue in surgeons [24]. The second time-out is therefore an opportunity for the surgeon to evaluate for progression of the operation as well as factors which are contributing to prolonged surgical duration.

Additionally, a second time-out can make the learning curve for robotic surgeries easier to overcome. Surgeons who are in the process of adopting robotic surgeries often explore the technology’s feasibility and limitations [25]. This second time-out gives attending surgeons a chance to both identify the challenges and limitations contributing to extended operative times and reconsider the need to either convert to a different approach or seek input from another surgeon.

Finally, while robotic surgeries can be less physically demanding than open or laparoscopic approaches, extended surgical times can still be physically and mentally taxing to both the attending surgeon and the surgical assistant. Indeed, two studies have shown that, despite decreased stress in robotic compared to laparoscopic surgery, the former is associated with poorer surgeon performance [26,27]. Thus, the second time-out is an opportunity for the surgical team to assess their mental and physical engagement, and the need for a brief break or change of surgical assistant.

#### Anesthesia considerations

Long duration surgeries are of particular anesthesia concern for several reasons. Not only is patient access limited once the robot arms are in place [28], but exaggerated Trendelenburg positioning and pneumoperitoneum have been shown to decrease venous outflow from the head, increase intraocular pressure, worsen ventilation-perfusion mismatch, and increase systemic catecholamine release [29]. Indeed, venous pooling in the head and neck during extended periods of Trendelenburg has resulted in laryngeal edema [9]. In these cases, fluid volumes must be carefully monitored to decrease the risk of post-operative edema [9,30]. Pneumoperitoneum also decreases pulmonary compliance, vital capacity, and functional residual capacity [31,32], increasing the risk of hemodynamic and acid–base alterations over time [33,34]. To this end, the second time-out provides a chance to assess the overall trend of urine output during the case while also reviewing the trend of vital signs for instances where there is a slow deterioration.

Blood loss should also be of increased concern during extended periods of Trendelenberg positioning due to the danger of posterior ischemic optic neuropathy (PION), among other complications. While the exact mechanism of PION is unclear, excessive blood loss, hypotension, increased venous pressure, and increased ocular pressure are proposed factors, all of which can be increased in robotic surgeries [35]. While blood loss should ostensibly be closely monitored throughout the case, the second time-out offers an opportunity to formally review both the trend in blood loss over the case and to discuss with the surgeon whether blood loss is excessive and what steps, if any, should be undertaken to address the issue. Furthermore, the second time-out is a good time to redose antibiotics and draw necessary labs. Reports have shown that redosing of antibiotics significantly decreases the rate of surgical site infections, especially in surgeries which last longer than 4 hours [36,37]. Redosing, however, may be forgotten in longer operations; only approximately one out of five cases with extended surgical times receive antibiotic redosing [37]. The second time-out addresses this issue by establishing a reminder for antibiotic administration.

#### Nursing considerations

Engagement of the nursing staff in the second time-out is important, as they are optimally situated to check patient positioning and padding. Additionally, the second time-out is an opportunity for the nursing staff to adjust equipment which they might not have time or access to check during the operation. Indeed, equipment malfunction can account for nearly 7% of surgical conversions in some surgeries [38], highlighting the importance of ensuring that all equipment is in working order during long and complex cases. While checking surgical equipment, care should be taken to re-check that sequential compression devices (SCD) are on correctly and working. Extended surgeries, especially ones lasting more than three hours, put the patient at increased risk for thromboembolic events [39,40], increasing the importance of pneumatic compression devices.

The second time-out is also an opportunity for nursing staff to verify surgical counts. Incorrect counts can be the most frequently reported adverse patient safety event during surgery [41], putting the patient at risk for multiple complications [42-44]. At the same time, case complexity and fatigue are significant risk factors for incorrect counts [44], putting surgeries with extended operative times at increased risk for incorrect surgical counts and highlighting the importance of a repeat count during the second time-out.

Lastly, the second time-out is a chance for nursing staff to update the OR administration on the room time. Not only does this give administrators time to ensure that the OR is adequately staffed with specialized robotic trained nurses, but it also allows for general operational planning.

## Conclusions

While robotic surgeries have many advantages, they also predispose to certain complications, especially those which stem from prolonged operative time. Simultaneously, longer robotic surgeries are becoming more prevalent as surgeons overcome the learning curve and attempt more complex cases. These long surgeries put a significant strain on nursing, anesthesia, and surgical staff in the OR. They also raise concerns which are often not an issue in shorter, non-robotic cases. To address these issues and facilitate inter-disciplinary communication, we have developed a comprehensive checklist to be conducted during a “second time-out” three to four hours after the beginning of the case.

At our institution, we have rolled out the second time-out on a preliminarily basis over the last year by asking surgeons to adopt it on a voluntary basis. A select group of surgeons utilizing the second time-out have expressed positive feedback so far. Our initial experience has demonstrated a relatively minimal intrusion to the case time for the second time-out to be performed (Can be as little as a few minutes to complete it). As such, we are continuing to accrue further feedback prior to proposing greater adoption. Ongoing studies are being performed to evaluate its effectiveness.

Future considerations regarding the second time-out include further check-points conducted at regular intervals after the second time-out has been conducted, as a way to ensure continued reflection and communication in cases which extend much further than 3–4 hours. Furthermore, based on the results of our ongoing studies regarding the effectiveness of the second time-out in robotic cases, this concept can potentially be applied to all prolonged surgeries.

## Competing interests

The authors declare that they have no competing interests.

## Authors’ contributions

JS contributed to acquisition of data, as well as analysis and interpretation of data. JS also was involved in drafting the manuscript with revisions critically for important intellectual content. GV and JM were involved in drafting the manuscript with revisions critically for important intellectual content. SB contributed to conception and design, analysis and interpretation of data. SB was also involved in drafting the manuscript and revising it critically for important intellectual content. All authors read and approved the final manuscript.
